# Clinical Profile, Risk Factors and Outcomes of Children With Cutaneous Larva Migrans Infection: A Hospital-Based Study

**DOI:** 10.7759/cureus.14416

**Published:** 2021-04-11

**Authors:** Vijayakumary Thadchanamoorthy, Kavinda Dayasiri

**Affiliations:** 1 Clinical Sciences Department, Faculty of Health Care Sciences, Eastern University, Batticaloa, LKA; 2 Internal Medicine-Pediatrics, Base Hospital, Mahaoya, LKA

**Keywords:** cutaneous larva migrans, children

## Abstract

Background

Cutaneous larva migrans (CLM) is a neglected parasitic skin disease caused by hookworm larvae such as *Ancylostoma braziliense* and *Ancylostoma caninum*. It is more common in tropical and subtropical countries. Evidence regarding clinical profiles, risk factors and outcomes of children with CLM in the Eastern province of Sri Lanka is scarce.

Aim

The aim of this study was to evaluate demographic profile, risk factors, clinical manifestations and outcomes associated with CLM in children who presented to paediatric and dermatological clinics of the Teaching Hospital, Batticaloa, Sri Lanka.

Method

A retrospective study was conducted over three years from January 2017 to December 2019 among children who presented with CLM infection to paediatric and dermatological clinics of the Teaching Hospital, Batticaloa, Sri Lanka. Twenty-eight children who were aged between 1 and 14 years were recruited for the study. Detailed information including demographic factors such as age, sex, residence and mother’s education, risk factors for infection, main reason for clinic visit, duration of illness, site of lesions, number of lesions, treatment received from out-patients department and/or general practitioner, duration of treatment, associated secondary bacterial infection, family history of similar infections and laboratory investigations were extracted from clinical records. Data were analyzed using SPSS version 19.0 (IBM Corp., Armonk, NY).

Results

Among 28 children recruited for this study, 19 (67.9%) were male children and 9 (32.1) were female children. The majority of children were below 6 years (n=25, 88.3%). Twenty-six (92.9%) children had residence in rural areas and also belonged to low socioeconomic class. Most cases were detected in Valaichenai (32.1%) and Kattankudy (21.4%) ‘Medical Officer of Health’ (MOH) regions. The majority of mothers were educated only up to ordinary level or below (n=19, 67.8%). All patients had pets either dogs or cats at home and no pets had been dewormed. Almost 92.9% of children acquired disease whilst playing on the infected soil

The main reason for clinic visit had been itchiness and this presentation accounted for 60.7% (n=17). Skin infection accounted for 25% of presenting problems. Majority of them presented late to the clinic with the lesions of more than three weeks of duration (n=17, 60.7%). Single lesion was noted in the majority (n=27, 96.4%) except one child who had three lesions. Buttock lesions were observed in 35.7%, feet in 25%, and 10.7% in legs.

Investigations revealed eosinophilia in 50% (n=14) of patients and neutrophil leukocytosis was seen in five patients (17.9%). All patients received treatment either from the general practitioner or outpatient department for variable duration without success before attending the specialist clinic. Fifty percent of patients needed treatment with Albendazole and antihistamine for more than three weeks to achieve complete cure. It was also observed that overall occurrence had been declining over the past three years.

Conclusion

CLM is a common and unreported disease in Eastern province, Sri Lanka. The majority of children presented from low socio-economic backgrounds. The common risk factors were the presence of dewormed pets at home and contamination with infected soil. The majority of children had a single lesion on presentation. Fifty percent of children needed more than three weeks of treatment to achieve a good response.

## Introduction

Cutaneous larva migrans (CLM) is a cutaneous dermatosis and more common in tropical and subtropical countries and also in countries where poor socioeconomic conditions prevail. It is caused by hookworms of dogs and cats, which include *Ancylostoma braziliense* and *Ancylostoma caninum* [1]. It is also known as creeping eruption as larvae enter the skin whilst children play on the infected soil, subsequently starting to creep and form linear lesions in the skin. It may manifest as either typical or atypical lesions. The atypical lesions are rare. The common lesions can be diagnosed clinically with their typical itchy, linear and erythematous tracts [2]. The common sites of infection are feet, thigh, perianal area and buttocks. Involvement of forearms and back of the chest was uncommon [3]. There are few systemic treatments available including albendazole or ivermectin which have been effective to achieve clinical cure. Topical treatment with albendazole has limited response as it needs to be applied for a long period [4]. The aim of this study was to evaluate demographic profile, risk factors, clinical information and outcomes associated with CLM in children who presented to paediatric and dermatological clinics of the Teaching Hospital, Batticaloa, Sri Lanka. 

## Materials and methods

A retrospective hospital-based study was conducted among children who attended the specialist paediatric and dermatology clinics of the Teaching Hospital, Batticaloa, Sri Lanka over three years from January 2017 to December 2019. Twenty-eight patients who were diagnosed with CLM and aged between one to fourteen years were analyzed. Only children in whom the diagnosis was verified and confirmed based on characteristic appearance of skin lesions either by consultant paediatrician or dermatologist were selected and recruited to the study. Specific diagnostic tests were not needed. Children with uncertain diagnoses were excluded from the study.

Detailed information including demographic factors include age, sex, residence, MOH area, socioeconomic status, mother’s education, duration of illness, main reason for clinic visit, site of lesion, number of lesions, treatment received from out-patients department or general practitioner, risk factors for infection, duration of treatment, associated secondary bacterial infection, family history of similar infections, regarding pets at home and laboratory investigations were extracted from clinical records. Socioeconomic status was assessed according to their monthly family income and a monthly income of less than 30 thousand Sri Lankan rupees and/ or obtaining Samurdhi allowances (an allowance given by the government for people with low family income) was defined as poor socioeconomic status. Eosinophilia has been defined as an eosinophil count of more than 500/cumm. In addition to evaluation of demographic data, the incidence of CLM infection was compared to assess trends over the three-year study period.

Data has been analyzed using SPSS version 19.0 (IBM Corp., Armonk, NY). Patient identifiable data were de-identified prior to accessing medical records. Written approval for the study was obtained from the Director, Teaching Hospital Batticaloa to retrieve clinic-based medical records.

## Results

Twenty-eight children were recruited to this study comprising 19 (67.9%) male and 9 (32.1) female. The majority of children had been below 6years (n=25, 88.3%) with most of them being aged 3 and 6 years (n=15, 53.6%). Age and sex distribution of the study population are shown in Table 1.

**Table 1:** Age and sex distribution of children with cutaneous larva migrans infection.

Age group	Male	Female	Total	Percentage (%)
≤3 yr	08	02	10	35.7
3+-6 yrs	09	06	15	53.6
6+-9 yrs	01	01	02	7.1
>9 yrs	01	00	01	3.6
Total	19	09	28	100

Regarding socioeconomic status, 26 (92.9%) patients have come from poor socioeconomic class and also rural areas and two patients (7.1%) came from high socioeconomic class and urban area.

Most of the patients visited from Valachenai (V) (32.1%) and Kattankudy area (K) (21.4%), respectively. Vakarai (VK) and Vavunathevu (VU) area had equal occurrences (10.7%) and Kiran (KI), Eravur (E) and Battticaloa (B) had 7.1% (n=2) of patients in each area. Only one patient (3.6%) visited from Chenkalady (C) area.

Out of 28 mothers, 19 (67.8%) mothers had studied only up to ordinary level or below. Eight (28.6%) mothers were educated up to advanced level and one mother (3.6%) was graduated from university. Only two (7.1%) mothers were employed while 26 (92.9%) mothers were housewives.

All (n=28, 100%) had pets in their homes either dogs or cats which had not been dewormed and 26 (92. 9%) patients reported playing on the soil within their home premises. One developmental delayed child had been regularly rolling over on their cemented visiting hall as he could not walk and, one other child had been doing circumrotation in their temple festival. No other family member was affected by similar illness.

The main reasons for clinic visit were severe itchiness (n=25, 89.3%), followed by skin eruptions due to secondary bacterial infection (n=7, 25%) and sleep disturbances (n=3, 10.7%). Pruritus anus and irritability had been noted in 7.1% (n=2) and 3.6% (n=1) of patients respectively.

This study showed that majority (n=17, 60.7%) had lesions for more than three weeks before visiting the specialist clinic while equal number of patients (n=5, 17.86%) presented with lesions in two weeks and three weeks duration. Least number (n=1, 3.6%) of them had a short duration of illness before they attended for specialist care.

Table 2 describes the location of lesions. The majority of lesions were found on buttocks (n=10, 35.7%) and feet (n=7, 25%). Three patients (10.7%) had lesions in legs. Two patients (7.1%) presented with perianal involvement. Here majority had a single lesion (n= 27, 96.4%) while one patient had three lesions (n=1, 3.6%).

**Table 2:** Pattern of distribution of skin lesions.

Site of the lesion	Number of children	Percentage (%)
Buttock	10	35.7
Perianal	02	7.1
Leg	03	10.7
Feet	07	25
Hand	02	7.1
Forearm	01	3.6
Abdomen	01	3.6
Chest	01	3.6
Buttock, dorsum of foot and sole	01	3.6

Investigation revealed eosinophilia in 75% (n=21) of patients whilst 25% (n=7) had normal blood counts. Although seven (25%) patients were suspected to have secondary infection, neutrophil leukocytosis was observed in only five patients (17.9%).

All had treatment either from general practitioner or outpatient department for a variable duration without success. Fifty percent (n=14) needed long-term treatment with appropriate dose of albendazole and antihistamine for three weeks or more. 28.6% (n=8) were given one-week treatment.

Over the three-year study period, the occurrence of CLM had been declining. Slight drop in occurrence had been observed in 2018 (35.7% of all children) from 2017 (42.9% of all children), but significant reductions were noticed in 2019 (21.4% of all children) as compared to the previous two years.

## Discussion

CLM is a neglected parasitic infestation in underprivileged communities in unindustrialized countries [1,5]. Though CLM is prevailing in our country, the burden of disease is often under-reported and few studies are performed to assess disease characteristics and outcomes. The diagnosis mainly depends on typical appearance of the disease, and the disease can potentially be missed or misdiagnosed by the medical practitioners. Previously, there were no studies performed on children with CLM in Eastern province of Sri Lanka. This study described the profile of the patients with CLM attended to specialist paediatric and dermatological clinics at Teaching Hospital, Batticaloa.

The study population in the current study comprised predominantly male children and the majority were below six years. Study done in rural community of Northeast Brazil [6] showed that it was more common in male than female and also 70% belong to less than nine years. There was another study in Brazil [7] which showed that male children were affected more than female children, but more among aged group between 10 to 14 years. All three studies showed males affected more than females. This might be that boys may spend more time in outdoors; have contact with soil while playing in the infected environment and parents might have showed attention less frequently. The current study showed that younger children were more affected than older children because they spent more time on playing in outdoors than elder children who spend more time on studying. Both boys and young children were affected more than other demographic groups in our study and this could be related to gender and age-related behavior.

This study highlighted that the majority of children who attended for treatment had poor socioeconomic status (n=26, 92.9%) and had been living in rural communities (n=26, 92.9%). All had pets that were not dewormed. All children had a history of either playing or rolling over soil. There were several studies done in rural and poor resource setting areas of other countries [6-8]. This could be that people living in urban areas are generally wealthy and mostly concern about the hygienic practices and although they have pets, they have been dewormed and children used sports shoes while playing. This cannot the situation in socio-economically deprived communities and also poverty-related living conditions like stray dogs and cats, unpaved streets and many unattended children playing in the contaminated soil create a beneficial environment for the larvae for transmission [6,9,10]. One child from urban and wealthy family acquired following circumrotation ritual in the temple where the environment would have been contaminated. Other child who had global developmental delay could only roll over on floor where cats were also lying and without deworming treatment.

Our study showed that there were more cases in Kattankudy (21.4%) and Valaichenai (32.1%) MO/MCH areas. This could be due to overcrowding in these two regions than other MO/MCH area. The study done in Brazil also had similar observations [8]. The educational levels of mothers were mostly ordinary level or below and accounted for 67.8%. Most of them were housewives (92.9%). Knowledge regarding personal hygiene, like wearing slippers and keeping the pets away from the environment where children play and deworming the pets would be accounted the education of the parents. A similar study in Brazil [7] concluded that health education might prevent CLM.

Our study revealed the main reasons for clinic visit was severe itchiness (n=25, 89.3%) and skin sepsis due to secondary bacterial infection (n=7, 25%) and sleep disturbances (n=3, 10.7%). The study done in Brazil [7] showed the sleep disturbance (81%) was the main reason for presentation among children followed by itchiness (61%) and super infection. Sleep disturbances could be due to intense itching. There were other two studies that described pruritus as the most common presenting symptom of CLM followed by sleep disturbances [11,12]. 

This study showed that the majority (n=17, 60.7%) had lesions for more than three weeks before visiting the specialist clinic and all had prior treatment from either general practitioner or outpatients’ department. This may be due to that they spend a lot of time for treatment outside and failures lead them to attend for expert opinion. The majority of lesions were found on the buttock (n=10, 35.7%) and feet (n=7, 25%). A study done in Brazil [7] and two other studies [13,14] reported that buttocks and feet would be the main regions affected and, this could be due to walking on barefoot and sitting on the infected soil. All patients in our study had one lesion except on child who had three lesions. The study in Brazil [7] showed average number of tracts per person is about one to three. This could be due to high prevalence of severe infestations in that region.

The current study showed eosinophilia in 50% (n=14) of patients with CLM. Although seven (25%) patients were suspected to have secondary bacterial infection, neutrophil leukocytosis was observed only in five patients (17.9%). As all patients were treated outside before presented to specialist clinic both with antibiotic and mebendazole, percentage of eosinophilia and evidence of super-infection were less evident. Study in Brazil showed that only 40% of their patients got treated outside before they presented to specialist clinic [7].

Majority of patients in our study (n=14, 50%) needed long-term treatment with appropriate dose of albendazole and antihistamine for three weeks or more. 28.6% (n=8) were given one-week treatment. One study [8] showed that 73.3% of patients with CLM required four-week treatment to obtain complete cure. This could be due to late presentation and longer duration of illness. Our study showed that overall incidence had been declining over the past three years from 2017 to 2019.

This study has several limitations. Given the low incidence of CLM infection, the study could recruit only 28 children for evaluation over the three-year study period. Limited sample size can potentially limit the strength of conclusions. Further, given the retrospective nature of study, only limited yet reliable data were extracted from medical records. Potential risk factors which were consistently recorded on case notes were considered for descriptive analysis. The authors suggest more rigorous analysis of prospectively collected potential risk factors in the logistic regression model to improve the strength of the observations. 

## Conclusions

CLM is a common and unreported disease in the Eastern province, Sri Lanka. The majority of children presented from low socio-economic backgrounds. The commonly reported potential risk factors were the presence of dewormed pets at home and contamination with infected soil. The majority of children had a single lesion on presentation. Fifty percent of children needed more than three weeks of treatment to achieve a good response.
